# Tuning the Porosity, Water Interaction, and Redispersion of Nanocellulose Hydrogels by Osmotic Dehydration

**DOI:** 10.1021/acsapm.1c01430

**Published:** 2021-12-22

**Authors:** Valentina Guccini, Josphat Phiri, Jon Trifol, Ville Rissanen, Seyede Maryam Mousavi, Jaana Vapaavuori, Tekla Tammelin, Thaddeus Maloney, Eero Kontturi

**Affiliations:** † Department of Bioproducts and Biosystems, Aalto University, P.O. Box 16300, 00076 Espoo, Finland; ‡ Department of Chemical and Metallurgical Engineering, School of Chemical Engineering, Aalto University, Kemistintie 1, 02150 Espoo, Finland; § VTT Technical Research Centre of Finland Ltd, VTT, PO Box 1000, FI-02044 Espoo, Finland; ∥ Department of Chemistry and Materials Science, Aalto University, Kemistintie 1, 02150 Espoo, Finland

**Keywords:** cellulose nanofibers, thermoporosimetry, ion conductivity, redispersion, controlled water removal

## Abstract

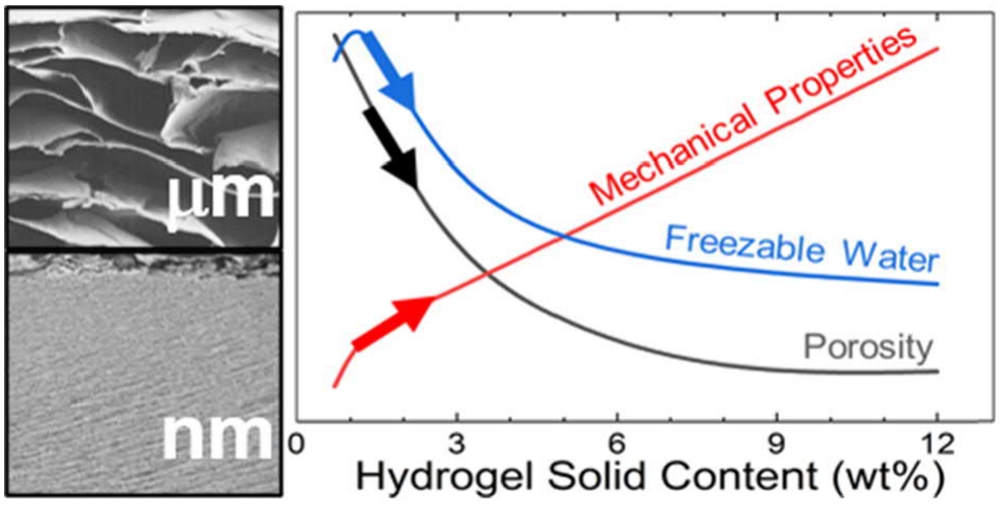

Osmotic dehydration (OD) was introduced as a method to reproducibly tune the water content and porosity of cellulose nanofiber (CNF) hydrogels. The hierarchical porosity was followed by electron microscopy (pores with a >100 μm diameter) and thermoporosimetry (mesopores), together with mechanical testing, in hydrogels with solid contents ranging from 0.7 to 12 wt %. Furthermore, a reciprocal correlation between proton conductivity and the ratio of water bound to the nanocellulose network was established, suggesting the potential of these systems toward tunable energy materials.

Nanocellulose is a class of naturally derived nanomaterials, offering excellent mechanical properties, biocompatibility, application range, and versatile possibilities for chemical modification coupled with biobased and renewable sourcing.^1−3^ This class encompasses cellulose nanocrystals (CNCs) and nanofibers (CNFs); the first are highly crystalline rigid rods, and the second are flexible semicrystalline filaments. Although their high water affinity can represent a challenge in composites and other applications targeted at load bearing, water retention is a significant asset for creating structural hydrogels.^2−6^ Because of their tendency to entangle, CNFs easily form viscoelastic solids (hydrogels) upon dehydration at concentrations as low as 0.25 wt %.^7^ When the water or solid content needs to be tuned, several methods are available to dehydrate hydrogels.^8^ Solvent evaporation poses the difficulty of precisely controlling the final solid content as time, heat, relative humidity, and ventilation all contribute to it. Moreover, it produces highly aggregated materials. Dehydration via filtration, spray drying, freeze-drying, and CO_2_ drying are time-consuming, complex, and costly^9−11^ and can induce the irreversible aggregation of the nanofibers, which decreases their redispersion and optical properties (e.g., transparency).^12^ Reproducible dehydration to a certain water content and control over the porosity in a hydrogel are also difficult with the listed methods. Such difficulties have most likely led to the fabrication of hydrogels by a bottom-up approach mostly limited to coagulating or cross-linking suspensions with a solid content of <2 wt %.^3,5^ CNFs and nanocellulose in general are particularly appealing compared to polymeric hydrogels in tissue engineering, the simulation of an extracellular matrix, wound healing, solid-state cell factories, and biomimicking applications as the 3D porous network can be tailored over a vast range of mechanical properties without losing its structural integrity.^2,13,14^ Especially for those applications involving living cells,^14^ the amount and type of porosity must be tailored for the specific biological proliferation as gas and fluid transport within the 3D structure define how suitable the material is for the specific application. In this respect, the fabrication technique plays a critical role. New bottom-up methods are needed to control the pore structure in order to increase the performance, aid the commercialization and promote the further material exploration of nanocellulose-based materials.^5,15,16^ Previously, Guccini et al.^17^ described the use of osmotic dehydration (OD) to precisely control the solid content of CNF suspensions (0.5 to 4.9 wt %) while minimizing the aggregation of the nanofibers.

In the present work, we demonstrate the untouched potential of osmotic dehydration (OD) as an innovative method to prepare hydrogels. For the first time we provide a detailed picture of the evolution of porosity in the wet state of the CNF hydrogels depending on their solid content (0.7 to 12 wt %) to which we correlate the conductivity, water physical characteristics, and mechanical properties. Additionally, we show that OD preserves the rheological properties of the CNFs upon rewetting. The carboxylated CNFs (Figure S1) used in this study were obtained by TEMPO-mediated oxidation.^18^Figure 1A schematically shows the OD setup used to manufacture the CNF hydrogels. The top compartment containing the CNFs was separated from the magnetically stirred PEG solution (35 kDa) by a semipermeable membrane (cut off 6–8 kDa), which allowed only the passage of water. Given the higher concentration of the PEG solution (10–25 wt %), water flows from the CNF to the PEG compartment by osmosis, thus dehydrating the CNF suspension. The rate of the diffusion can be tuned by the PEG concentration and thus the dehydration can be easily controlled and performed at room temperature and atmospheric pressure. OD leads to homogeneous and smooth hydrogels whose dimensions can be potentially scaled up (Figure S2). Figure 1B shows that the solid content increases exponentially from 0.7 ± 0.1 to 12.4 ± 0.8 wt % depending on the PEG concentration, which corresponds to a decrease in the moisture content. Despite the pivotal role that the dehydration plays in nanocellulose assembly and rheological and mechanical properties, this effect has rarely been systematically correlated with the physical and chemical properties of the derived hydrogels, such as porosity and water interaction.^8,12^ This can be achieved using OD. Figure 1C shows the freeze-dried cross sections of two of the hydrogels imaged by scanning electron microscopy (SEM). At 0.7 wt %, the hydrogel possesses very large pores in the range of ca. 100 μm, and these pores are separated by walls that are a couple of micrometers thick. At 12 wt %, the cross section appears very compact and dense, with pores in the mesoporous range. A high-resolution SEM image of a single-wall structure is featured in the Supporting Information (Figure S3). Their thicknesses are also different, ≈400 mm at 0.7 wt % and ≈20 mm at 12 wt %. Despite the large difference in the solid content, both cross sections show the typical layered structure, suggesting that its formation happens progressively during dehydration. We note that the characterization of the wet (or swollen) porosity from SEM imaging may not be totally accurate because of the artifacts that can be produced during the freeze-drying of the sample.

**Figure 1:**
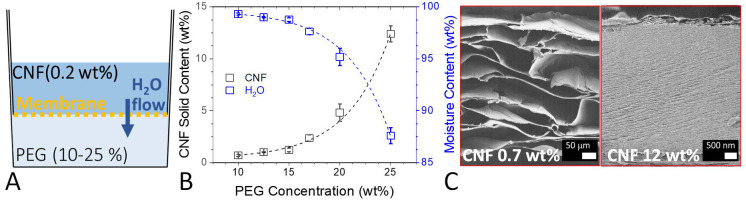
(A) Schematic representation of the osmotic dehydration setup, (B) CNF solid and moisture content of the hydrogels as a function of the PEG concentration, and (C) SEM images of the cross sections of the freeze-dried hydrogels prepared using 10 and 25 wt % PEG.

Thermoporosimetry measures the porosity directly in the wet state^19−21^ and recently has been successfully employed to characterize pore size distribution and water characteristics in cellulosic fibers^22,23^ as well as in CNC-based systems.^24,25^Figure 2A shows the wet porosity of the CNF hydrogels as a function of the CNF solid content. During the dehydration, the distance between the nanofibers decreases, creating an interconnected 3D network that provides the porosity in the wet state. The total pore volume stays relatively constant at around 17 mL g^–1^ between 0.7 and 1.2 wt % solid contents. Once the solid content reaches 12 wt %, the total pore volume decreases to a minimum of 3.9 ± 0.7 mL g^–1^. Most of the pores are in the mesoporous range, accounting to ≈97% at the lowest CNF concentration and ≈99% at the highest. The pore size distribution (PSD) of the hydrogels is shown in Figure 2B. The peak of each PSD is above 10 nm in all cases and decreases from ≈20 to 16 nm as the solid content increases. While the values from the mesoporous range based on the melting point depression are deemed as accurate, thermoporosimetry can only offer an estimation of the macroporosity;^21^ therefore, the micrometer-sized pores visible from the SEM images are not accounted for in the distribution. This means that the large (∼100 μm) pores visible in the SEM images of the low weight percent hydrogels (Figure 1C, left-hand side) are not accounted for in the pore volume (Figure 2A) and PSD (Figure 2B) analyses by thermoporosimetry. In other words, only the pores inside the walls of the low weight percent hydrogels are determined in Figure 2A and B. Nevertheless, a clear trend within the >100 nm pore region can be seen. The number of macropores between 50 and 100 nm sharply decreases with the increase in the solid content. These results provide a detailed picture of the porosity evolution in the wet state of the hydrogels as a function of their solid content. CNF films prepared by vacuum filtration have shown a pore volume in the wet state three orders of magnitude lower but have also shown larger pore diameters (≈ 30 nm).^21^ These differences most likely originate from nanofiber aggregation induced by filtration during drying, which may also explain why the pore dimensions are constant despite 200 h of swelling in water. By carefully modulating the porosity via OD, it is possible to tune the water interactions as well as the liquid and gas uptakes of the hydrogels. Indeed, the macropores aid the fast water uptake and swelling but allow low water penetration within the solid structure, while the mesopores aid the slower water absorption but larger penetration via capillary forces.^3^ Briefly, the water in nanocellulose matrices can be defined as free and bound water with respect to its thermodynamic properties, which are influenced by the effect of the confinement within the matrix and the strong nanocellulose–water interaction.^26^ “Free water” has the same chemical and physical properties as bulk water and it is chiefly responsible for the swelling of the CNF matrix. “Bound freezing water” is confined and weakly bound in the submicroscopic space created from the CNF network. Such confinement affects its solid–liquid phase transition. “Non-freezing water” is tightly bound at the cellulose surface and does not freeze due to strong interaction. Figure 2C shows the percentage of bound and free water with respect to the total amount of freezing water. Below 1.2 wt % the ratio is relatively even, after which the amount of free water decreases while the amount of bound water increases at higher solid contents. At 12 wt % the share of bound water is nearly 90% of the total water. We stress that contrary to Figure 2A and B, the larger pores in the low weight percent hydrogels, as visualized by SEM (Figure 1C, left-hand side), are accounted for as free water because all frozen water is considered here. The increase in the amount of bound water with the CNF solid content relates to the decrease of porosity in the hydrogels (Figure 2A and 2B), which ultimately provides more confinement for the water and thus changes in its thermodynamic properties. The fact that the porosity is relatively stable below 1.2 wt % but the moisture content increases (Figure 1B) suggests that the free water is capable of swelling the hydrogel structure, increasing its porosity only to a certain limit (up to 1.2 wt %), and the excess water is likely phase-separated and loosely retained within the CNF network.

**Figure 2:**
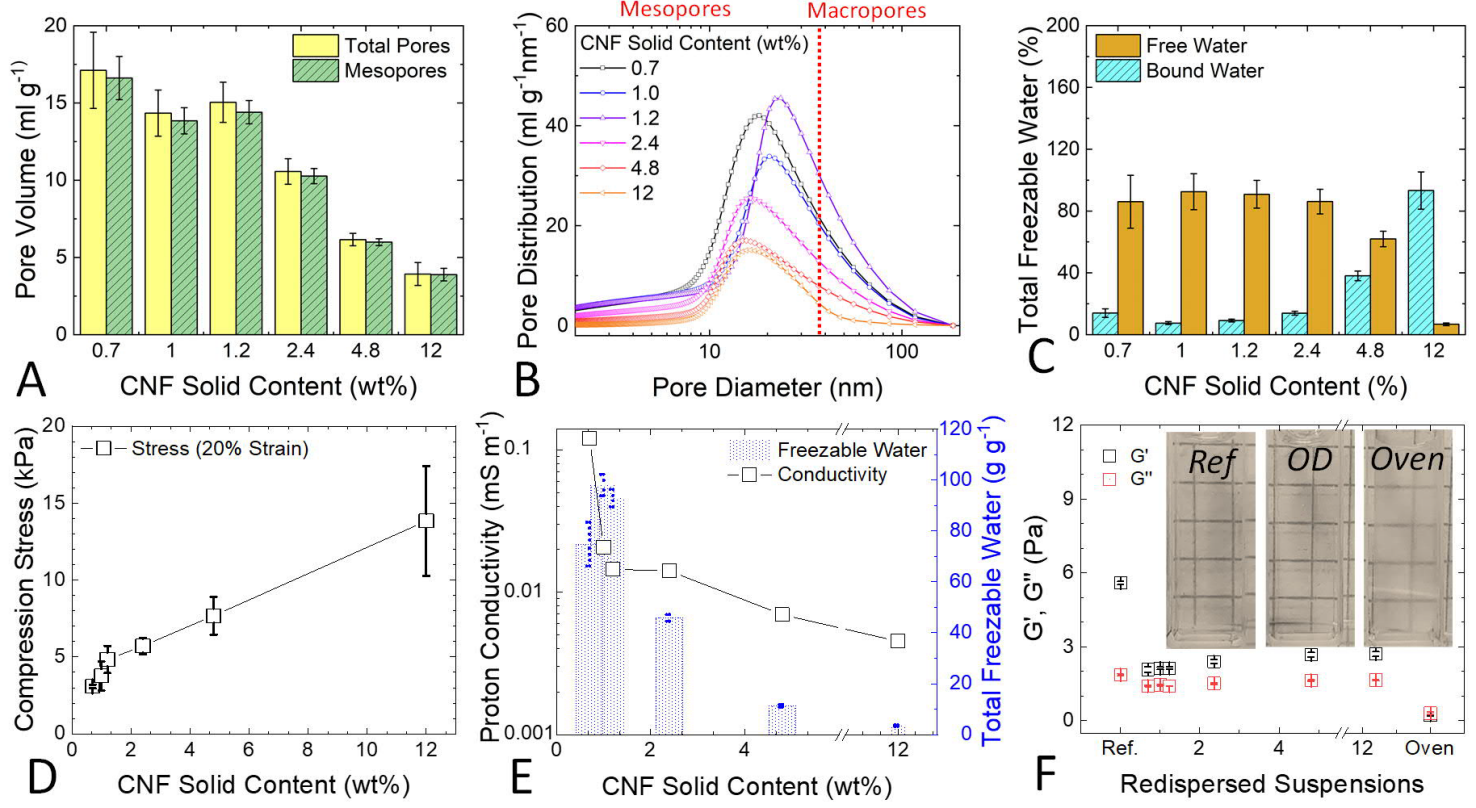
(A) Pore volume, (B) pore size distribution, and (C) percentage of freezable water of the hydrogels analyzed by thermoporosimetry. (D) compression stress and (E) proton conductivity of the hydrogels in correlation with the amount of freezable water per gram of CNF. (F) Storage (*G*′) and loss (*G*″) modulus of the initial hydrogels before dehydration (ref) and after (0.7–12 wt %) OD or oven drying (Oven). The inset show an optical comparison of the redispersed hydrogels (OD and Oven) with the original suspension (ref).

The variation in mechanical properties of the hydrogels was analyzed by a compression test. Figure 2D shows that the stress increases with the CNF solid content, meaning that the hydrogels become harder to deform. The rate at which the stress increases is higher below 1.2 wt %. This is related with the fact that the deformations due to water loss and densification are more pronounced because of the higher moisture content and porosity (Figures 1B and 2A). At higher solid contents, the larger amount of bound water is clearly less prone to be lost compared to the free water due to its strong association with the CNFs (Figure 2C). This factor and the lower porosity make the hydrogel stronger. Moreover, the behavior of CNFs at the nanoscale also contributes. According to our previous study,^17^ above 1.0 wt % the nanofiber-entangled network goes through an isotropic–anisotropic transition (formation of nematic order), leading to stronger yet stiffer materials. The reliable control of the solid content offered by OD can be used to gain insight into the fundamental properties of water within the CNF 3D network. Figure 2E shows the proton conductivity of the hydrogels compared to the freezable water. The conductivity drops nearly 10× from 0.7 to 1.0 wt % and is ≈100 times lower above 2.4 wt %. Since very little research has been conducted on the relationship between the proton conductivity and the amount and state of the water molecules in nanocellulose systems,^6^ our easily controllable system is ideal to elucidate this question. Here, we found a strong correlation with the amount of water and likely with the porosity. As the porosity and the amount of free water sharply decrease above 2.4 wt % (Figure 2A and C), the conductivity drops another order of magnitude compared to that at 0.7 wt %. This shows the predominant role of free water over the bound water in the conductivity, which occurs via water-mediated hydrogen bonding (Grötthuss mechanism). These results highlight that bound water cannot sufficiently conduct protons. These observations also have direct practical implications if these gels are considered as, for example, membranes whose applicability toward proton exchange member fuel cells are being assessed.^27^ Interestingly, electrochemical conditions within, for example, a fuel cell may affect the conduction properties of bound water, as has been suggested by the fact that carboxylate CNF membranes have shown constant conductivity above 65% relative humidity.^28^ As previously demonstrated, water removal with OD reduces the aggregation of CNF.^17^ Thus, we evaluate OD as a method to improve the redispersion of concentrated CNFs. After OD, the hydrogels were redispersed to the initial concentration (0.2 wt %) simply by magnetic stirring overnight. The dispersions appeared to be stable over a period of some weeks. Figure 2F shows the comparison of storage (*G*′) and loss (*G*″) moduli in the linear viscoelastic region between the initial dispersions, the redispersed OD samples, and the redispersed oven-dried (to >95 wt %) 105 °C sample. After the OD, the solid-like character (*G*′ > *G*″) of the suspensions was preserved, although the elastic behavior and ability of the CNF network to dissipate stress without irreversible deformation decreased, as can be seen from the lower *G*′ values and early yield behavior (onset of nonlinear behavior and critical stress), respectively (Figure S7A). This is most likely connected to the formation of small aggregates that disrupt the entanglement network within the suspension. Nevertheless, OD clearly minimizes CNF aggregation, as can also be seen in the inset of Figure 2F from the unchanged appearance of the redispersed hydrogels (OD) compared to that of the pristine suspension (ref). The absence of large aggregates was also confirmed by thermal analysis (Figure S7B). In contrast, *G*′ of the oven-dried samples decreases more than 10× with values lower than *G*″, indicating irreversible aggregation during drying that is also evident from the sample turbidity (Figure 2F inset). While several strategies have been implemented for CNCs, CNFs are significantly more difficult to redisperse due to their inherent tendency to entangle.^29,30^ Although it is difficult to compare rheological data in general due to the many factors affecting the measurements,^7^ our findings show very good redispersion with very little energy input. This could be further improved, for example, by introducing additives during the water removal or simply increasing the shear force of the mixing during redispersion.^30^

## Conclusions

Ultimately, our study offers a platform to regulate fluid and gas transport in nanocellulose hydrogels. Because of the reliable control of the solid content offered by OD, we were able to elucidate the relationship between porosity, water physical characteristics, and mechanical properties, which are also critical for other nanocellulose based-materials such as membranes, films, and foams. The analyzed hydrogels (0.7–12 wt %) have a hierarchical porosity in the meso- and macroporous range. The total pore volume stays relatively constant up to 1.2 wt %, above which it sharply decreases. At the same time, the amount of free water decreases and the amount of bound water increases. We found out that the free water has a crucial role in the proton conduction mechanism. Finally, OD minimized the aggregation of the nanofibers, which allowed the redispersion of the hydrogels in water without a loss of their elastic character (*G*′ > *G*″).

## Supporting Information Available

Additional experimental details, including additional microscopy images and photographs of new samples, and raw data for compression tests, thermoporosimetry, and proton conductivity (PDF)
