# Supplementary material for: Tuning the Porosity, Water Interaction, and Redispersion of Nanocellulose Hydrogels by Osmotic Dehydration
Source: ACS Appl Polym Mater. 2021 Dec 22;4(1):24–8. doi: 10.1021/acsapm.1c01430 (PMC8765005; doi:10.1021/acsapm.1c01430)
Supplement: Supplementary file 1 — ap1c01430_si_001.pdf [file ap1c01430_si_001.pdf]

Supporting Information

Tuning the porosity, water interaction and redispersion of nanocellulose hydrogels by osmotic dehydration

Valentina Guccini,[a] Josphat Phiri,[a] Jon Trifol,[b] Ville Rissanen,[c] Maryam Mousavi,[d] Jaana Vapaavuori,[d] Tekla Tammelin,[c] Thaddeus Maloney[a] and Eero Kontturi*[a]*

a. Department of Bioproducts and Biosystems, Aalto University, P.O. Box 16300, 00076, Espoo, Finland

b. Department of Chemical and Metallurgical Engineering, School of Chemical Engineering, Aalto University, Kemistintie 1, 02150 Espoo, Finland

c. VTT Technical Research Centre of Finland Ltd, VTT, PO Box 1000, FI-02044 Espoo, Finland

d. Department of Chemistry and Materials Science, Kemistintie 1, 02150 Espoo, Finland

* Corresponding authors: valentina.guccini@aalto.fi; eero.kontturi@aalto.fi

Table of contents

Preparation and characterization of cellulose nanofibres……………………………….S1

Osmotic dehydration…………………………………………………………………….S2

Characterization of the hydrogels………………………………………………………S3

Redispersibility of osmotically dried hydrogels………………………………………..S5

References………………………………………………………………………………...S7

1. Preparation and characterization of cellulose nanofibers

TEMPO-mediated oxidation and fibrillation. The nanofibers were produced from never-dried cellulose pulp of bleached softwood obtained from a Finnish pulp mill, consisting of a mixture between spruce and pine. The TEMPO-mediated oxidation of the pulp was carried out following the protocol describe by Saito et al.,[1] using hypochlorite in alkaline conditions. After the reaction, the oxidized pulp was washed and redispersed in water to a final concentration of ca. 1 wt%. The fibrillation process was carried out twice at 1850 bar, using a microfluidizer

(Microfluidics Int., USA) equipped with two Z-type chambers with diameters of 400 and 100 µm. The final solids content of the CNF suspension was 0.89 wt% which was measured by evaporating the excess water overnight at 105 °C in a ventilated oven.

Surface charge. The carboxylate content of the CNFs was measured with a standard conductimetry titration method (SCAN 65:02) on the oxidized pulp. The anionic charge was 1.47 mmol g^{-1}.

Morphology. The nanofibers were imaged by Atomic Force Microscope in tapping mode, model Bruker Instrument (minus KR Tech.), using Ultrasharp µmarch silicon tips (HQ: NSC15/Al BS, Tallinn, Estonia) characterized by a typical force constant of 40 N m^{-1}. The nanofibers were deposited on silicon substrate coated with PEI via spin coating (1.5 min at 4000 rpm). **Figure S1** shows an image of the CNFs with a height (width) of 3-4 nm and lengths running up to micrometres.

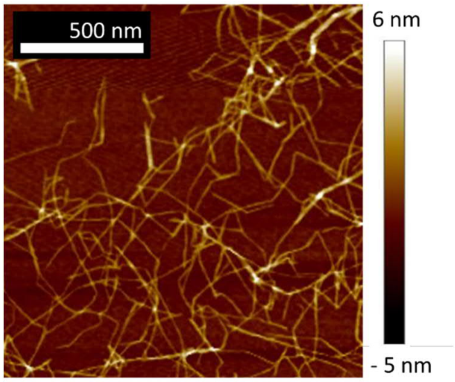

Figure S1. AFM image of the CNFs

2. Osmotic dehydration (OD)

PEG solutions were prepared simply by mixing the proper amount of solid PEG with water to obtain the desired wt%. The suspension was then magnetically stirred until the complete dissolution of PEG. The CNF hydrogels were prepared with a grammage of 0.639 mg cm^{-2}. Firstly the 0.89 wt% CNF suspension was diluted to 0.3 wt% and homogenised at 12.4 rpm using Turrax. An aliquot of 12.6 g was taken and further diluted to 0.2 wt% and mixed by vortex. The air trapped in the suspension was removed by vacuum, after which the nanofiber suspension was gently poured into the top compartment of the OD set-up (including Spectra/por Dialysis Membranes from regenerated cellulose, molecular weight cut-off 6 to 8 kDa). The bottom compartment was filled with the same amount of PEG solutions (15.6 ml)

with the appropriate concentration (25, 20, 17, 15, 12.5 and 10 wt%) and stirred with a magnetic bar. The dehydration was carried out for 24 hours at room temperature. The 24 h dehydration time was chosen from a set of robust trial-and-error type of experiments where we noticed that the wt% was always stable after 24 h. Depending on the characterization technique the samples were coagulated by immersion in a 0.1 M HCl solution for 10 min, after which they were washed until neutral pH and stored in aqueous solution. HCl coagulation was used for the samples that underwent mechanical testing and proton conduction measurement, because exchanging the carboxylic counterion to proton provided better mechanical strength and enabled measuring the proton conduction. In fact, the gels with low solids contents were too weak to handle for, e.g., the compression tests. By contrast, the thermoporosimetry measurements and redispersion experiments (rheology) were done right after the osmotic dehydration, i.e. without HCl coagulation. In fact, redispersion of CNFs was not feasible when the carboxylic groups were protonated. Moreover, the excess water was difficult to remove from the HCl-treated gels, and this would have affected the amount of free water analyzed by thermoporosimetry. The solid content of the hydrogels was obtained by drying the samples right after the dehydration in the oven at 105 ˚C for 24 hours. The weight was recorded before and after with a balance with five significant digits. The measurement was repeated 3 times.

3. Characterization of the hydrogels

The hydrogels obtained by OD are homogenous and smooth; **Figure S2** shows three of them.

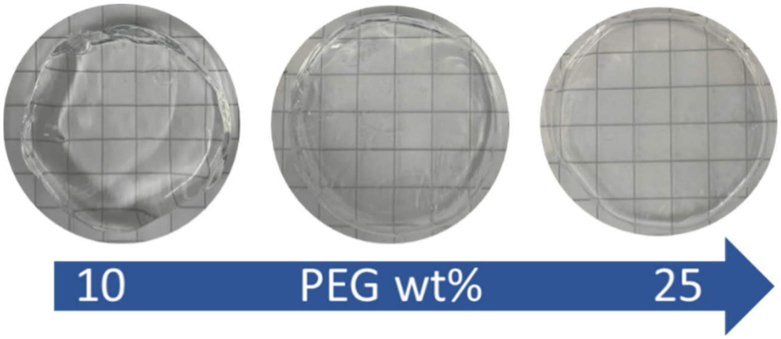

Figure S2. Images of the CNF hydrogels (diameter 3.8 cm) prepared using 10, 15 and 25 wt% of PEG solutions (left to right)

Microscopic characterization. A JEOL JSM-7500FA analytical scanning electron microscope (SEM) was used to image the hydrogel porosity. The hydrogels were frozen and freeze dried. The freezing was done in a plastic bag to minimize the eventuality of directional

freezing induced by the container (e.g., metal). The cross-section was prepared by cooling the samples with liquid nitrogen and fracture it with a scalpel. Prior to image with SEM all the samples were coated with a 2 nm thick layer of Au/Pd.

Figure S3 shows a complementary image to the ones in Figure 1C. Here, a higher resolution image from a 0.7 wt% hydrogel is shown, i.e., the same sample as in Figure 1C (left hand side). It reveals the compact structure of a thin (~2-3 µm) single wall separating the large, ~100 µm pores.

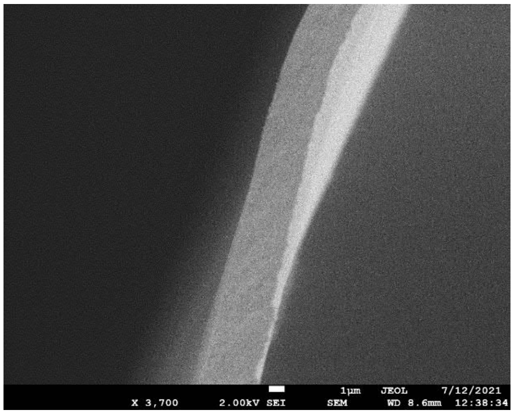

Figure S3. High resolution SEM image from a single wall separating the large (~100 µm) pores, as depicted in Figure S1C (left hand side) image in the main manuscript.

Porosity and water interaction. Thermoporosimetry was used to accurately measure the porosity of the hydrogels in wet state. The measurements were done on a Mettler Toledo DSC 3+ (Mettler-Toledo Intl. Inc. Instrument, USA) differential scanning calorimeter (DSC) equipped with an intracooler. The samples were hermitically sealed in 40 µL aluminium pans. The masses of the sealed crucibles were recorded before and after the measurements to ensure that the Al pans remained sealed during the measurement. The temperature was first brought to –50 °C at 20 K min^{-1} to crystalize all the freezable water in the samples. The temperature was then increased to –0.2 °C and held constant until the melting transition was completed i.e., until all the confined water melted. This step is essential to prevent supercooling during the subsequent recrystallization step. The temperature was then decreased at 2 K min^{-1} to –50 °C. The freezing bound water (FBW) was calculated by integrating the resulting exothermic peak. In the next step, the temperature was increased to +60 °C at 10 K min^{-1} and the total freezable water (TFW), sum of bound and free water, was calculated by integrating the resulting endothermic. A typical DSC curve of the hydrogel with 12 wt% CNF is shown in **Figure S4A**.

The TFW is shown in **Figure S4B** and **Figure S4C** shows the cumulative pore volume of the hydrogels.

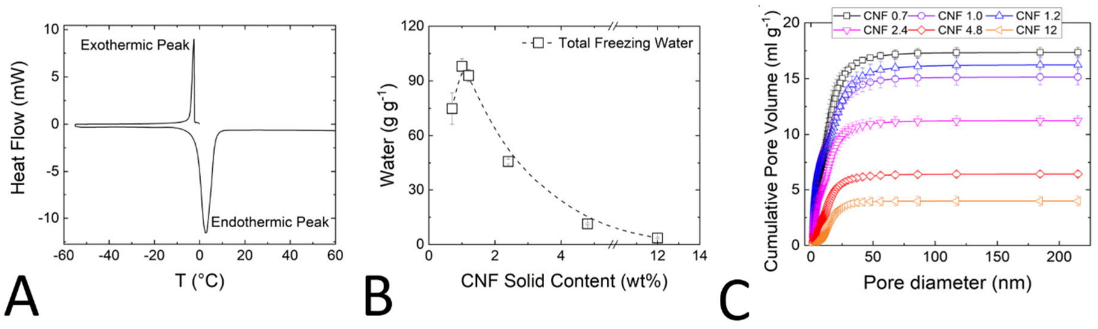

Figure S4. **(A)** DSC curve of the hydrogel with 12 wt% CNF. **(B)** Total Freezable water calculated during melting up to 60 °C. **(C)** Cumulative Pore volume of the CNF hydrogels

Mechanical properties. The compression test was carried out with a Dynamic Mechanical Analyzer (TA Q800, Instrument, New Castle, DE, US) using 3 mm plates and a 3 mm thick aluminium bed. The coagulated hydrogels were cut to 29 mm diameter using a sample cutter, after which they were placed in the chamber and equilibrated at 25 °C for 5 minutes. After the equilibration, the samples were subjected to controlled compression force up to 18 N with 4 N min^{-1} rate. The compression test was done on at least three replicas. **Figure S5** shows a typical compression curve of the samples.

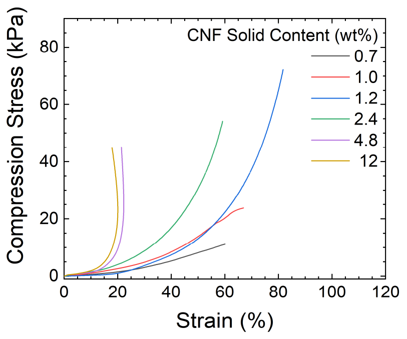

Figure S5. Typical stress-strain curve of the hydrogels

Proton Conduction. The through plane proton conductivity of the hydrogels was measured by Electrochemical Impedance Spectroscopy (EIS) using a Ivium Vertex Potentiostat, (The Netherlands). The experimental set up was built according to the work of Müller et al.[2] The frequency range of 100 KHz to 100 mHz and the amplitude of 10 mV were used, which guarantee noise free measurements. Prior to the measurements the hydrogels were cut parallel

to the cross-section using a 10 mm blade to a thickness of 320 ± 0.9 µm. The thickness was measured using a Sony IMX219 camera (12.3 Megapixels), according to the procedure reported in literature.[3] Experimental data were fitted using Randles circuit (inset in Figure S5). The ionic conductivity of the samples was calculated using Equation 1,

$$\sigma = \frac{L}{R_p A} \qquad \textbf{eq.1}$$

in which σ is the ionic conductivity, L represents thickness of the samples, R_p and A are ionic resistance and contact area between the electrodes and the hydrogels. The impedance curves are shown inf **Figure S6**.

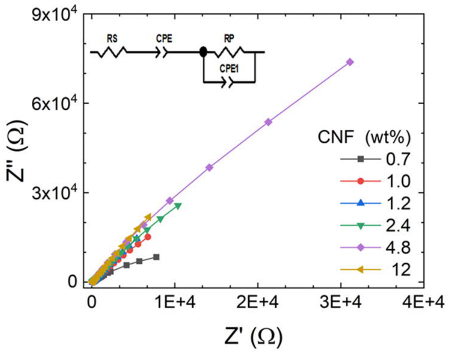

Figure S6. Impedance curves of the CNF hydrogel from which the conductivity was calculated. Inset: Randles Circuit is in which *Rs* represents the bulk resistance, R_p the ion diffusion resistance and CPE the constant phase element.

4. Redispersibility of the osmotically dried hydrogels

After the 24 hours of dehydration, the hydrogels (0.7 – 12 wt%) had been directly redispersed in deionized water up to the initial concentration of 0.2 wt%, after which they were gently stirred at 128 rpm with a magnetic bar for 24 hours.

Rheological characterization. The measurements were performed at 22 °C using an AR-G2 rheometer (TA Instruments, New Castle, United States) equipped with plate–plate geometry (diameter 40mm). The viscoelastic properties of the 0.2% CNF suspension before and after osmotic dehydration were measured by oscillatory stress and frequency sweep tests. The stress sweep tests were performed between 0.005 and 100 Pa under a constant frequency of 1 Hz, and the frequency sweep tests between 0.05 and 50 Hz at a constant stress of 0.05 Pa (linear viscoelastic region). Triplicate samples of the hydrogels were analyzed for each measurement,

and all samples were allowed to relax for 2 minutes under the measuring head prior to the measurements. G' and G'' were obtained from the linear viscoelastic region in the stress curves. Onset of nonlinear behavior (ONL) was defined as the first G' data point to deviate over 5% from the linear region. Critical stress (CS) was obtained from stress-strain curves, as the first point where the strain begins to exhibit nonlinear behavior (over 10% deviation from slope = 1). Both ONL and CS are shown in **Figure S7A**.

Contribution of nanofiber aggregation. The redispersed hydrogels were centrifuge at 4500 rpm for 1 hour. The supernatant (1 ml) was collected, and its solid content was compared to the one prior centrifugation. Both were measured by a thermogravimetric analyser (TGA), model Q500 from TA Instruments, using a heating rate of 10 °C min^{-1} up to 105 °C and isotherm of 10 minutes. The standard deviation of this method was measured on triplicates. The samples were compared to the suspension that was not dried at all and to the one dried at 105 °C in the oven. The difference between the concentrations before the centrifuge and the top 1 ml supernatant gives a measure of the redispersion degree since the eventual presence of aggregates at the bottom of the centrifuge tube will deplete the nanofiber concentration. This difference is shown in **Figure S7B**.

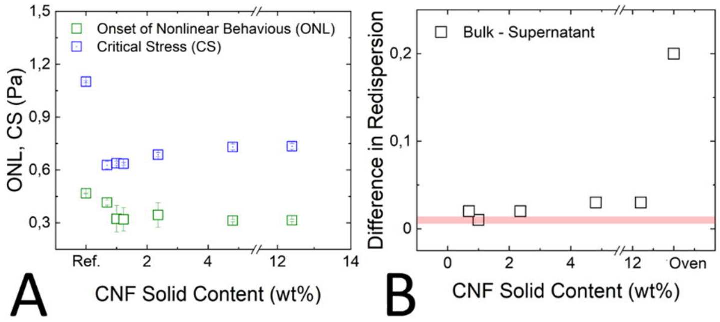

Figure S7. **(A)** Onset of the nonlinear behaviour and critical stress of CNF suspensions before (*Ref.*) and after OD. **(B)** Comparison of the redispersion degree based on the difference in weight. The red line represents the standard deviation of the method.

All values of hydrogels prepared by OD lay very close to the standard deviation of the measurements (red line), meaning that the difference is just above the method uncertainty. Thus, all OD samples show very good redispersion and very small aggregation. Instead, the data scatter with the sample prepared by oven drying is significantly higher, indicating strong aggregation and worse redispersion.

References

(1) Isogai, A.; Saito, T.; Fukuzumi, H. TEMPO-Oxidized Cellulose Nanofibers. *Nanoscale* **2011**, *3* (1), 71–85. https://doi.org/10.1039/C0NR00583E.

(2) Müller, F.; Ferreira, C. A.; Azambuja, D. S.; Alemán, C.; Armelin, E. Measuring the Proton Conductivity of Ion-Exchange Membranes Using Electrochemical Impedance Spectroscopy and Through-Plane Cell. *J. Phys. Chem. B* **2014**, *118* (4), 1102–1112. https://doi.org/10.1021/JP409675Z.

(3) Miikki, K.; Karakoç, A.; Rafiee, M.; Lee, D. W.; Vapaavuori, J.; Tersteegen, J.; Lemetti, L.; Paltakari, J. An Open-Source Camera System for Experimental Measurements. *SoftwareX* **2021**, *14*, 100688. https://doi.org/10.1016/j.softx.2021.100688.
